# Predictors of mammographic density among women with a strong family history of breast cancer

**DOI:** 10.1186/s12885-019-5855-2

**Published:** 2019-06-26

**Authors:** Olivia Moran, Andrea Eisen, Rochelle Demsky, Kristina Blackmore, Julia A. Knight, Seema Panchal, Ophira Ginsburg, Kevin Zbuk, Martin Yaffe, Kelly A. Metcalfe, Steven A. Narod, Joanne Kotsopoulos

**Affiliations:** 1 Women’s College Research Institute, Women’s College Hospital, 76 Grenville St., 6th Floor, Toronto, ON Canada; 2 Department of Nutritional Sciences, University of Toronto, Toronto, ON Canada; 3 Toronto-Sunnybrook Regional Cancer Center, Toronto, ON Canada; 4 Division of Gynecologic Oncology, Princess Margaret Hospital, University Health Network, Toronto, ON Canada; 5 Department of Molecular Genetics, University of Toronto, Toronto, ON Canada; 6 Prevention and Cancer Control, Cancer Care Ontario, Toronto, ON Canada; 7 Lunenfeld-Tanenbaum Research Institute, Sinai Health System, Toronto, ON Canada; 8 Dalla Lana School of Public Health, University of Toronto, Toronto, ON Canada; 9 Mount Sinai Hospital, University of Toronto, Toronto, ON Canada; 10 Perlmutter Cancer Centre, Department of Population Health, NYU Langone Health, New York, NY USA; 11 Department of Oncology, McMaster University, Hamilton, ON Canada; 12 Department of Medical Biophysics, Sunnybrook Research Institute, University of Toronto, Toronto, ON Canada; 13 Lawrence S. Bloomberg Faculty of Nursing, University of Toronto, Toronto, ON Canada

**Keywords:** mammographic density, breast cancer, risk factors, familial breast cancer

## Abstract

**Background:**

Mammographic density is one of the strongest risk factors for breast cancer. In the general population, mammographic density can be modified by various exposures; whether this is true for women a strong family history is not known. Thus, we evaluated the association between reproductive, hormonal, and lifestyle risk factors and mammographic density among women with a strong family history of breast cancer but no *BRCA1* or *BRCA2* mutation.

**Methods:**

We included 97 premenopausal and 59 postmenopausal women (age range: 27-68 years). Risk factor data was extracted from the research questionnaire closest in time to the mammogram performed nearest to enrollment. The Cumulus software was used to measure percent density, dense area, and non-dense area for each mammogram. Multivariate generalized linear models were used to evaluate the relationships between breast cancer risk factors and measures of mammographic density, adjusting for relevant covariates.

**Results:**

Among premenopausal women, those who had two live births had a mean percent density of 28.8% vs. 41.6% among women who had one live birth (*P*=0.04). Women with a high body weight had a lower mean percent density compared to women with a low body weight among premenopausal (17.6% vs. 33.2%; *P*=0.0006) and postmenopausal women (8.7% vs. 14.7%; *P*=0.04). Among premenopausal women, those who smoked for 14 years or longer had a lower mean dense area compared to women who smoked for a shorter duration (25.3cm^2^ vs. 53.1cm^2^; *P=*0.002). Among postmenopausal women, former smokers had a higher mean percent density (19.5% vs. 10.8%; *P*=0.003) and dense area (26.9% vs. 16.4%; *P*=0.01) compared to never smokers. After applying the Bonferroni correction, the association between body weight and percent density among premenopausal women remained statistically significant.

**Conclusions:**

In this cohort of women with a strong family history of breast cancer, body weight was associated with mammographic density. These findings suggest that mammographic density may explain the underlying relationship between some of these risk factors and breast cancer risk, and lend support for the inclusion of mammographic density into risk prediction models.

**Electronic supplementary material:**

The online version of this article (10.1186/s12885-019-5855-2) contains supplementary material, which is available to authorized users.

## Background

Mammographic density remains one of the strongest predictors of breast cancer risk among women in the general population [1]. Women with high percent density (≥75% density) have an estimated 4.6-fold increased risk of breast cancer compared to women with low percent density (<5% density) [1]. While the biological basis underlying this relationship is unclear, high mammographic density may reflect the cumulative exposure of the dense breast stroma and epithelium to hormonal and growth factors, which can stimulate their proliferation and lead to carcinogenesis [2, 3]. A family history of breast cancer, which is also a strong risk factor for the disease, has been shown to be associated with higher mammographic density in addition to greater breast cancer risk [4–6].

Along with family history, several reproductive, hormonal and lifestyle risk factors for breast cancer are also associated with mammographic density, suggesting that mammographic density may mediate the relationships between some of these risk factors and breast cancer risk [2, 7]. To our knowledge, there is very little literature evaluating whether breast cancer risk factors are associated with mammographic density among high-risk populations, including no studies among *BRCA* mutation carriers and only one study among women with a family history [8].

Therefore, it is not established whether the reproductive, hormonal, and lifestyle breast cancer risk factors that modify mammographic density in the general population are applicable to women with a strong family history of breast cancer. Given the evidence suggesting that mammographic density is strongly implicated in familial breast cancers [4–6, 8], evaluating whether these risk factors are associated with mammographic density may help explain the underlying mechanisms mediating their relationships with breast cancer risk among high-risk women. Thus, the goal of the current study was to evaluate the relationship between various reproductive, hormonal, and lifestyle risk factors with three measures of mammographic density (e.g., percent density, dense area, and non-dense area), among women with a strong family history of disease but no *BRCA* mutation.

## Methods

### Study Population

Potentially eligible subjects were identified from a longitudinal prospective cohort study of women with a strong family history of breast cancer but no identified *BRCA* mutation, based at Women’s College Hospital (Toronto, Canada) and previously described in detail [9]. *BRCA* mutation detection in the affected relatives was performed using a variety of genetic testing methodologies, as techniques have evolved over time, but all nucleotide sequences were confirmed by Sanger direct sequencing. Pedigrees were retrospectively reviewed to identify families that met the eligibility criteria. The probands (i.e., the first family member to undergo genetic testing) of the eligible families were invited to share the study information with their cancer-free, first-degree relatives.

Participants were recruited from six genetic counselling clinics in Ontario. Participants were eligible if 1) there was no *BRCA* mutation found in their affected relatives; 2) they had a strong family history of breast cancer; and 3) they had no personal history of cancer or a prophylactic bilateral mastectomy prior to enrollment. A strong family history entailed two breast cancers in first-degree relatives under the age of 50, or three cases of breast cancer at any age on the paternal or maternal side of the family. This study was approved by the institutional ethics review boards of the six collaborating institutions and all participants provided written informed consent.

### Data Collection

Upon enrollment into the overarching cohort study, participants complete a self-administered baseline research questionnaire, a Diet History Questionnaire (DHQ) [10], and biological samples were collected [9]. Participants signed a medical release form for review of their breast imaging and pathology reports. A follow-up questionnaire was mailed biennially to all subjects in order to update their personal medical histories and to ascertain incident disease.

The research questionnaires collected detailed information regarding family history, reproductive histories, preventive surgeries and breast screening, and lifestyle factors such as physical activity, smoking and alcohol consumption. Specifically, the questionnaires inquired about parity (number of live births), duration of breastfeeding, exogenous hormone use (oral contraceptives and hormone replacement therapy), height and weight, the average number of alcoholic drinks consumed per week (0-3, 4-9, 10-20 and 20 or more), and smoking history (number of packs of cigarettes smoked per week). ‘Ever’ alcohol drinkers were defined as those who reported consuming at least one alcoholic beverage within the last six months.

Briefly, physical activity was ascertained using a check-list originally developed for the Nurses’ Health Study [11] and has been previously validated [12]. Participants reported the length of time (ranging from 0 to ≥11 hours) they spent engaged in various physical activities per week during the last 12 months, including: walking, jogging, swimming, tennis, aerobics and weight lifting. A Metabolic Equivalent of Task (MET) score was assigned to each physical activity to describe its intensity, using criteria established by Ainsworth *et al.,* [13]. MET values for walking were determined based on the reported walking pace (average: 3.0 METs). A total weekly activity score was calculated by summing all MET values for each participant. In order to investigate the independent effect of moderate-to-vigorous physical activity (MVPA), a second physical activity score, that included only activities at the moderate and vigorous intensity levels, was also created.

### Subjects Available for Analyses

Women were included in the current study if they were between the ages of 18-65 years at enrollment and reported undergoing at least one mammogram. Medical release forms were used to obtain mammographic images. For women who reported undergoing mammography at their baseline research questionnaire, the mammogram closest to the completion date of this questionnaire was requested. For participants who had their first mammogram after their baseline questionnaire, the follow-up questionnaire closest in time to this mammogram was requested. Of the 191 participants who reported having at least one mammogram, mammographic images from 168 participants were successfully obtained. Mammograms that were not compatible with the Cumulus software due to compressed formatting were excluded (n = 11), thus 157 participants were included in the final analysis. Only screening mammograms were requested, thus, none of the participants had a breast cancer diagnosis at the time of, or prior to, the mammograms used in this analysis. The majority of women (n = 131) had undergone mammography at the time of their baseline questionnaire. Follow-up questionnaires were used for women who initiated mammography screening after the completion of their baseline questionnaire (First follow-up: n = 17; Second follow-up: n = 8; Third follow-up: n = 1).

### Mammographic Density Assessment

The Cumulus software (University of Toronto, Toronto, Canada) was used to quantify mammographic density. Cumulus is a semi-automated, computer-assisted thresholding method and is the gold standard for mammographic density assessment [14]. Mammographic density is strongly correlated between breasts [15], thus the cranio-caudal view of the right breast of each subject was used to measure percent density. To determine the dense area and non-dense area, the pixel spacing of each image (range: 49 to 252 μm/pixel) was converted into centimetres squared (cm^2^) to determine the area of each pixel. Next, the dense area and total breast area were multiplied by the pixel area. The non-dense area was determined by subtracting the dense area by the total breast area. Thirteen of the 157 mammographic images were films, and nine were previously digitized by the original imaging centres. The remaining four films were digitized by the study team using a pixel spacing of 252 μm/pixel and the iCAD scanner and Fulcrum software.

The mammographic images were read in three batches by two readers. Reader 1 (MY) read Batch 1 and Batch 2 (number of images [n] Batch 1: n = 61; Batch 2: n = 39) and Reader 2 (OM) read Batch 3 (n = 57). In all three batches, the images were randomized and 15% of the images were randomly repeated. There was high reproducibility within each batch, yielding a mean within-batch correlation coefficient of 0.91. For all images that were repeated, the average value of the mammographic density measures were used. An accurate pixel size could not be obtained for a subset of the images (n = 13), thus dense area and non-dense area could not be assessed.

### Statistical Analyses

All exposures were analyzed continuously and dichotomously *a priori* using high vs. low categories based on the median distribution in the cohort. The 75^th^ percentile was used for the anthropometric variables because of their strong correlation with mammographic density. Given the low levels of physical activity in this group of women, the 75^th^ percentiles for the physical activity variables were used to maximize the investigation of high physical activity and mammographic density. Percent density, dense area, and non-dense area were non-normally distributed; therefore, all values were square root-transformed to improve normality. We excluded one postmenopausal woman with a percent density value of 77.2%, since it was four standard deviations above the mean. Thus, 59 postmenopausal women were included in the analyses.

Analyses were stratified by menopausal status *a priori* because mammographic density declines after menopause [3]. Differences in descriptive characteristics were evaluated using the Student’s *t*-test for continuous variables and the χ^2^-square test for categorical variables. Generalized linear models were used to obtain the β-estimates, least-square adjusted means, and the 95% confidence intervals (CI) of percent density, dense area, and non-dense area. The least-square adjusted means and corresponding 95% CI were back-transformed to facilitate interpretation of findings. All models adjusted for age and BMI at the time of the mammogram and parity. Since the mammograms came from different sources, all models were adjusted for modality (digital mammograms, films digitized by the original imaging centers, and films digitized by the study team). The models for total live births and breastfeeding were further adjusted for age at first live birth. The height and weight models were mutually adjusted for each other instead of BMI. The physical activity and alcohol models were additionally adjusted for smoking status (never, former, current). The smoking models were additionally adjusted for alcohol consumption (drinks per week). Single imputation was performed for missing quantitative data points by imputing the median value of the exposure. This was done for age at menarche (number of missing data points [n] = 2) and age at first use of alcohol (n = 1). For any missing qualitative data (i.e., never/ever), we attempted to contact study subjects to retrieve the missing information, after which any remaining data was left as missing. Analyses were conducted using SAS version 9.3 (SAS Institute, Cary, NC, USA).

All *P* values were based on two-sided tests. We applied the Bonferroni correction to account for the multiple comparisons and given that some comparisons involved groups with counts less than five (*P* ≤ 0.0002, based on 222 tests and α = 0.05). This calculation includes the statistical analyses performed in the Additional file 1: Table S1, Additional file 2: Table S2, Additional file 3: Table S3, and Additional file 4: Table S4.

## Results

A total of 97 premenopausal and 59 postmenopausal women were included in the current study (Table 1). Postmenopausal women were older (*P* <0.0001), had a lower mean percent density (*P* <0.0001), a lower mean absolute dense area (*P* = 0.0001), and a greater mean non-dense area (*P* = 0.04) compared to premenopausal women. On average, postmenopausal women had a greater mean number of live births (2.3 births vs. 1.9 births; *P* = 0.005) and were younger at their first live birth compared to premenopausal women (25.5 years vs. 30.2 years; *P* <0.0001). Among smokers, postmenopausal women smoked a greater number of packs of cigarettes per week compared to premenopausal women (4.2 packs vs. 2.8 packs; *P* = 0.01).

**Table 1:** Characteristics at baseline of study population stratified by menopausal status

Characteristic	Premenopausal Women *N* = 97	Postmenopausal Women *N* = 59
Age at questionnaire, mean (range)	43.3 (27.0-58.3)	56.6 (43.2-68.3)
Age at mammogram, mean (range)	43.9 (29.2-59.0)	56.5 (41.8-68.2)
Years between questionnaire and mammogram, mean (range)	0.9 (0-5.6)	0.6 (0-2.0)
Percent Density (%), mean (range)	27.5 (0.2-79.7)	14.0 (0.7-58.3)
Dense Area (cm^2^), mean (range)	33.2 (0.6-152.8)	18.4 (1.6-111.8)
Non-Dense Area (cm^2^), mean (range)	116.1 (16.6-496.1)	143.0 (50.7-348.5)
Height (cm), mean (range)	166.5 (149.9-188.0)	164.0 (152.0-175.3)
BMI (kg/m^2^), mean (range)		
Current	25.8 (18.0-48.0)	26.7 (18.4-42.0)
At 18 years	21.4 (15.7-44.3)	20.4 (16.9-26.6)
At 30 years	23.7 (18.0-46.3)	22.3 (17.7-34.9)
At 40 years	24.3 (18.8-42.9)	24.2 (17.7-38.6)
Greatest BMI	27.2 (18.3-49.6)	28.5 (20.6-42.0)
Age at menarche, mean (range)	12.8 (8.0-16.0)	13.1 (11.0-16.0)
Parity^1^, *n* (%)	71 (73)	46 (77)
Nulliparous	26 (27)	14 (23)
1	21 (30)	6 (13)
2	35 (49)	22 (48)
≥3	15 (21)	18 (39)
Mean (range)	1.9 (1-3)	2.3 (1-4)
Age at first birth, mean (range)	30.2 (18.0-42.0)	25.5 (15.0-37.0)
Breastfeeding, mean months (range)^2^	15.1 (0-87)	9.3 (0-39)
Age at menopause,^3^ mean (range)	N/A	46.4 (26-56)
Hormone therapy use, *n* (%)^3^		
Never	N/A	43 (73)
Ever		16 (27)
Years of HRT use, mean (range)		6.8 (0.3-23.0)
Oral contraceptive use, *n* (%)		
Never	10 (10)	5 (9)
Ever	87 (90)	53 (91)
Missing	0	1 (2)
Years of OC use, mean (range)	9.6 (0.5-34.0)	9.6 (0.08-30.0)
Alcohol use, *n* (%)		
Never	10 (10)	5 (8)
Ever	87 (90)	55 (92)
Current	73 (79)	46 (84)
Drinks consumed per week, mean (range)	3.9 (2-15)	4.7 (2-20)
Age of initiation, mean (range)	18.2 (14.0-30.0)	18.8 (13.0-40.0)
Smoking history, *n* (%)		
Never	64 (66)	30 (51)
Ever	33 (34)	29 (49)
Current	7 (20)	7 (22)
Number of packs smoked per week, mean (range)	2.8 (1-7)	4.2 (1-9)
Age of initiation, mean (range)	17.4 (13.0-29.0)	16.1 (11.0-23.0)
Physical Activity Within the last year		
Total physical activity MET^4^-hours per week, mean (range)	36.2 (0-162.6)	30.5 (0-211.0)
MVPA^5^ MET-hours per week, mean (range)	23.3 (0-147.6)	19.1 (0-141.0)

^1^Parity includes live births only

^2^Among parous women only

^3^Among postmenopausal women only

^4^Metabolic Equivalent of Task. Includes walking and moderate and vigorous physical activity

^5^Moderate to vigorous physical activity

Overall, the mean time difference between the mammogram and the questionnaire used was 0.9 years (range: 0-5.6 years) for premenopausal women and 0.6 years (range: 0-2.0 years) for postmenopausal women (*P* = 0.01). No other differences were observed between the two groups (*P* ≥ 0.06).

### Reproductive and Hormonal Risk Factors

Among parous premenopausal women (n = 71), increasing number of live births was modestly associated with lower percent density (β = -0.60; 95% CI -1.26, 0.06; *P* = 0.07) (Additional file 1: Table S1). Women who had two live births had an adjusted mean percent density of 28.8% compared to 41.6% among women who had one live birth (*P* = 0.04) (Table 2). Among parous postmenopausal women (n = 46), women who had two or more live births had higher adjusted mean non-dense areas of 143.0 cm^2^ and 146.4 cm^2^, respectively, compared to 95.1 cm^2^ among women who had one live birth (both *P* = 0.04) (Table 3). No other significant associations were observed between the reproductive and hormonal exposures among premenopausal or postmenopausal women (*P* ≥ 0.06) (Tables 2 and 3**,** Additional file 1: Table S1 and Additional file 2: Table S2).

**Table 2:** Adjusted mean mammographic density measures according to reproductive and hormonal exposures among premenopausal women

		Premenopausal women (*n* = 97)										
		Percent Density (%)				Dense Area (cm^2^)				Non-Dense Area (cm^2^)		
	*n*	Mean^1^ PD	95% CI^1^	*P* ^*1*^	*n*	Mean^1^ DA	95% CI^1^	*P* ^*1*^	*n*	Mean^1^ NDA	95% CI^1^	*P* ^*1*^
Age at menarche												
<13 years	32	29.9	20.0-41.8		28	31.1	17.2-49.2		28	80.1	50.4-116.7	
≥13 years	65	28.2	19.6-38.3	0.69	60	35.3	22.5-51.1	0.49	60	98.2	69.8-131.6	0.16
Parity												
Nulliparous	26	32.5	21.5-45.8		23	37.1	21.4-57.0		23	93.6	60.5-133.8	
Parous	71	28.1	19.7-38.1	0.33	65	33.8	21.3-49.2	0.61	65	93.9	66.1-126.6	0.98
Total live births^2^												
1	21	41.6	27.2-59.0	ref	19	50.1	28.0-78.6	ref	19	71.7	39.0-114.3	ref
2	35	28.8	17.8-42.3	0.04	32	32.9	17.2-53.7	0.06	32	84.7	52.2-125.0	0.40
≥3	15	29.1	15.3-47.3	0.12	14	33.0	13.7-60.8	0.14	14	82.5	42.6-135.3	0.58
Breastfeeding^2^												
Never	4	34.9	13.2-67.0		4	44.1	13.7-91.8		4	77.9	26.9-155.3	
Ever	66	31.7	20.7-45.1	0.79	61	36.0	19.8-56.9	0.64	61	81.5	50.7-119.6	0.90
Breastfeeding duration^2^												
<11 months	30	32.7	18.7-50.5		31	41.9	21.2-69.6		31	76.5	41.4-122.4	
≥11 months	40	31.7	20.6-45.2	0.87	34	34.9	18.9-55.8	0.43	34	82.8	51.3-121.8	0.69
OC use												
Never	10	29.6	17.5-44.9		9	33.2	15.8-57.1		9	72.4	38.4-117.2	
Ever	87	28.5	19.9-38.7	0.87	79	34.5	21.9-50.0	0.89	79	97.3	69.1-130.3	0.18
Duration of OC use												
<9 years	40	37.4	25.2-52.0		37	41.5	24.6-62.8		37	79.9	50.0-116.8	
≥9 years	47	33.0	22.1-46.0	0.32	42	35.9	20.4-55.9	0.39	42	86.1	54.9-124.2	0.60

^1^Adjusted least-square means, 95% confidence intervals, and *P*-values are from analyses using square root-transformed mammographic density measures. The adjusted least-square means and 95% confidence intervals were back-transformed

^2^Among parous women only

All models were adjusted for age (continuous) and BMI (continuous) at the time of mammogram, parity (continuous), and mammogram modality (digital image, film scanned by study team, film scanned by imaging centre). The parity (total live births) and breastfeeding models were additionally adjusted for age at first birth (continuous). OC, oral contraceptives

**Table 3:** Adjusted mean mammographic density measures according to reproductive and hormonal exposures among postmenopausal women

		Postmenopausal women (*n* = 59)										
		Percent Density (%)				Dense Area (cm^2^)				Non-Dense Area (cm^2^)		
	*n*	Mean^1^ PD	95% CI^1^	*P* ^*1*^	*n*	Mean^1^ DA	95% CI^1^	*P* ^*1*^	*n*	Mean^1^ NDA	95% CI^1^	*P* ^*1*^
Age at menarche												
<13 years	21	14.3	8.7-21.2		18	18.0	10.8-27.0		18	121.6	92.3-154.9	
≥13 years	38	13.8	8.6-20.2	0.85	37	21.7	14.2-30.8	0.36	37	133.0	104.3-165.3	0.44
Parity												
Nulliparous	13	17.4	10.3-26.2		11	24.9	15.1-37.1		11	120.0	86.8-158.6	
Parous	46	13.5	8.6-19.4	0.24	44	19.4	12.7-27.5	0.25	44	128.1	101.4-158.0	0.63
Total live births^2^												
1	6	21.3	11.7-33.8	ref	6	29.4	17.8-44.0	ref	6	95.1	59.5-139.1	ref
2	22	12.9	7.0-20.4	0.09	20	19.5	12.0-28.9	0.11	20	143.0	106.9-184.3	0.04
≥3	18	12.8	6.4-21.5	0.11	18	20.9	12.3-31.6	0.19	18	146.4	106.4-192.9	0.04
Breastfeeding^2^												
Never	10	14.1	6.3-25.0		10	26.4	15.2-40.6		10	154.9	106.5-212.4	
Ever	35	14.2	8.3-21.8	0.97	34	20.9	13.5-29.8	0.29	34	129.6	97.0-166.8	0.25
Breastfeeding duration^2^												
<11 months	26	12.9	6.9-20.8		24	20.9	12.8-31.1		24	148.8	110.8-192.5	
≥11 months	20	15.8	8.9-24.7	0.41	20	22.7	14.2-33.3	0.69	20	119.8	85.8-159.4	0.12
OC use												
Never	5	9.0	2.6-19.3		4	16.4	5.5-33.2		4	149.4	94.9-216.2	
Ever	53	13.9	9.2-19.7	0.24	50	20.1	13.4-28.1	0.59	50	128.9	103.3-157.3	0.45
Duration of OC use												
<9 years	26	14.6	9.0-21.5		26	19.9	12.4-29.2		26	120.5	93.5-151.0	
≥9 years	27	12.9	7.6-19.6	0.55	24	20.1	12.3-29.8	0.95	24	140.6	110.3-174.6	0.16
HRT use												
Never	43	13.7	8.7-19.7	ref	39	19.9	13.1-28.1	ref	39	133.0	106.5-162.5	ref
Former	9	14.8	7.2-25.0	0.77	9	19.1	9.4-32.1	0.88	9	100.4	66.3-141.5	0.08
Current	7	16.1	7.4-28.2	0.60	7	24.0	11.7-40.8	0.52	7	123.8	80.7-176.1	0.68

^1^Adjusted least-square means, 95% confidence intervals, and *P*-values are from analyses using square root-transformed mammographic density measures. The adjusted least-square means and 95% confidence intervals were back-transformed

^2^Among parous women only

All models were adjusted for age (continuous) and BMI (continuous) at the time of mammogram, parity (continuous), and mammogram modality (digital image, film scanned by study team, film scanned by imaging centre).The parity (total live births) and breastfeeding models were additionally adjusted for age at first birth (continuous). OC, oral contraceptives. HRT, hormone replacement therapy

### Anthropometric Risk Factors

Among premenopausal women, an increasing body weight was associated with significantly lower percent density (β = -0.05; 95% CI -0.07, -0.03; *P* <0.0001) and significantly greater non-dense area (β = 0.13; 95% CI 0.09, 0.17; *P* <0.0001) (Additional file 3: Table S3). Women with a high body weight had a significantly lower adjusted mean percent density (17.6% vs. 33.2%; *P* = 0.0006) and a significantly greater adjusted mean non-dense area (165.2 cm^2^ vs. 73.2 cm^2^; *P* <0.0001) than women with a low body weight (Table 4). Increasing height was associated with significantly lower non-dense area (β = -0.12; 95% CI -0.21, -0.03; *P* = 0.01) (Additional file 3: Table S3). Among postmenopausal women, an increasing body weight was associated with lower percent density (β = -0.05; 95% CI -0.07, -0.02; *P* = 0.001) and higher non-dense area (β = 0.13; 95% CI 0.08, 0.18; *P* <0.0001) (Additional file 4: Table S4). Women with a high body weight had a significantly lower adjusted mean percent density (8.7% vs. 14.7%; *P* = 0.04) and a greater adjusted mean non-dense area (184.8 cm^2^ vs. 117.4 cm^2^; *P* = 0.0007) compared to women with a low body weight (Table 5). Women with a greater height had a significantly lower adjusted mean non-dense area of 98.3 cm^2^ compared to 137.3 cm^2^ among women with a lower height (*P* = 0.01) (Table 5).

**Table 4:** Adjusted mean mammographic density measures according to anthropometric and lifestyle factors among premenopausal women

		Premenopausal women (*n* = 97)										
		Percent Density (%)				Dense Area (cm^2^)				Non-Dense Area (cm^2^)		
	*n*	Mean^1^ PD	95% CI^1^	*P* ^*1*^	*n*	Mean^1^ DA	95% CI^1^	*P* ^*1*^	*n*	Mean^1^ NDA	95% CI^1^	*P* ^*1*^
Weight												
<77.0 kg	71	33.2	23.7-44.2		64	35.3	22.4-51.1		64	73.2	47.6-104.4	
≥77.0 kg	26	17.6	9.4-28.4	0.0006	24	31.9	17.1-51.4	0.59	24	165.2	115.9-223.2	<0.0001
Height												
<170 cm	69	27.0	18.3-37.5		63	32.4	19.2-48.9		63	97.7	66.5-135.0	
≥170 cm	28	33.2	23.1-45.1	0.18	25	37.0	23.1-54.3	0.47	25	78.6	51.3-111.8	0.16
TWA												
<43.0 MET-hrs/week	69	26.4	17.1-37.8		62	30.6	17.4-47.5		63	94.6	63.0-132.6	
≥43.0 MET-hrs/week	28	24.1	14.6-36.0	0.58	26	29.8	15.7-48.2	0.88	25	93.9	59.8-135.6	0.96
MVPA												
<27.0 METs/week	72	26.1	17.1-37.0		64	30.2	17.3-46.6		64	93.9	63.0-131.0	
≥27.0 METs/week	25	23.6	13.6-36.3	0.55	24	30.7	16.0-50.1	0.93	24	95.6	60.2-139.3	0.90
Smoking status												
Never	64	28.9	20.3-39.1	ref	58	33.5	20.8-49.3	ref	58	89.5	61.4-122.9	ref
Former	26	29.3	19.3-41.5	0.93	23	36.2	21.5-54.8	0.68	23	102.4	68.7-142.9	0.36
Current smoker	7	18.1	6.8-34.9	0.14	7	19.7	5.2-43.5	0.15	7	87.7	42.3-149.3	0.94
Age at first use												
<16 years	10	19.8	7.3-38.3		10	30.0	12.3-55.4		10	130.4	78.6-195.3	
≥16 years	23	28.8	14.0-49.0	0.15	20	46.7	25.0-75.1	0.07	20	127.9	79.8-187.4	0.90
Packs smoked												
<3 packs/week	20	29.2	14.4-49.3		18	48.1	27.5-74.5		18	125.5	78.4-183.6	
≥3 packs/week	13	19.5	7.3-37.6	0.12	12	25.5	10.0-48.2	0.01	12	135.9	81.7-203.8	0.64
Duration of use												
<14 years	16	32.2	17.4-51.3		14	53.1	32.0-79.5		14	126.0	77.5-186.3	
≥14 years	17	15.8	5.6-31.2	0.01	16	25.3	11.0-45.5	0.002	16	132.7	80.6-197.8	0.76
Alcohol use												
Never	10	29.7	17.4-45.1	ref	9	38.1	18.9-63.8	ref	9	103.3	61.0-156.6	ref
Former	15	30.5	18.1-46.2	0.91	15	32.8	16.4-54.7	0.64	15	79.7	45.8-122.9	0.31
Current user	72	27.9	19.1-38.3	0.78	64	33.8	21.0-49.7	0.66	64	96.0	67.3-129.7	0.73
Age at first use												
<18 years	29	21.5	11.8-34.2		27	28.9	15.2-47.0		27	118.8	78.4-167.6	
≥18 years	58	27.5	17.7-39.3	0.14	52	34.3	21.0-51.0	0.34	52	90.6	60.1-127.4	0.05
Drinks consumed												
<2 drinks/week	60	25.2	15.4-37.4		57	33.0	19.1-50.7		57	92.5	59.0-133.4	
≥2 drinks/week	27	27.7	17.0-41.1	0.58	22	33.2	19.0-51.3	0.98	22	101.1	65.3-144.6	0.58

^1^Adjusted least-square means, 95% confidence intervals, and *P*-values are from analyses using square root-transformed mammographic density measures. The adjusted least-square means and 95% confidence intervals were back-transformed

All models were adjusted for age (continuous) and BMI (continuous) at the time of mammogram, parity (continuous), and mammogram modality (digital image, film scanned by study team, film scanned by imaging centre). The height and weight models were mutually adjusted for each other (continuous) instead of BMI. The physical activity models were additionally adjusted for smoking status (never, former, current). The smoking models were additionally adjusted for the number of alcoholic drinks consumed per week (continuous). The alcohol models were additionally adjusted for smoking status (never/ever). TWA, total weekly activity. MVPA, moderate-to-vigorous physical activity. METs, Metabolic Equivalent of Task

**Table 5:** Adjusted mean mammographic density measures according to anthropometric and lifestyle factors among postmenopausal women

		Postmenopausal women (*n* = 59)										
		Percent Density (%)				Dense Area (cm^2^)				Non-Dense Area (cm^2^)		
	*n*	Mean^1^ PD	95% CI^1^	*P* ^*1*^	*n*	Mean^1^ DA	95% CI^1^	*P* ^*1*^	*n*	Mean^1^ NDA	95% CI^1^	*P* ^*1*^
Weight												
<77.0 kg	43	14.7	9.3-21.5		40	19.4	12.4-27.8		40	117.4	88.0-151.0	
≥77.0 kg	16	8.7	4.2-14.8	0.04	15	18.7	11.3-28.1	0.88	15	184.8	143.4-231.4	0.0007
Height												
<170 cm	47	13.4	8.8-19.1		43	20.3	13.8-28.2		43	137.3	111.7-165.6	
≥170 cm	12	15.4	8.2-24.7	0.56	12	18.3	9.4-30.0	0.63	12	98.3	66.9-135.7	0.01
TWA												
<43.0 MET-hrs/week	49	13.7	8.7-19.7		45	18.9	12.1-27.2		45	123.5	96.3-154.1	
≥43.0 MET-hrs/week	10	12.7	6.4-21.0	0.76	10	19.5	10.4-31.6	0.88	10	148.7	108.4-195.3	0.16
MVPA												
<27.0 METs/week	45	12.4	7.6-18.3		41	17.0	10.5-25.1		41	128.8	99.4-162.1	
≥27.0 METs/week	14	15.8	9.5-23.6	0.25	14	23.1	14.3-33.9	0.14	14	126.5	93.3-164.7	0.88
Smoking status												
Never	30	10.8	6.5-16.1	ref	29	16.4	10.3-23.9	ref	29	138.6	110.4-170.1	ref
Former	22	19.5	13.2-27.0	0.003	20	26.9	18.1-37.4	0.01	20	108.4	81.1-139.8	0.04
Current smoker	7	10.8	4.5-19.9	0.99	6	14	5.4-26.4	0.62	6	134.0	89.4-187.7	0.84
Age at first use												
<16 years	14	20.5	9.7-35.3		12	32.0	15.9-53.6		12	91.2	47.1-149.8	
≥16 years	15	26.2	14.8-40.8	0.35	14	36.9	21.9-55.8	0.58	14	111.3	68.8-163.9	0.42
Packs smoked												
<3 packs per week	8	18.8	7.9-34.3		7	35.0	17.0-59.4		7	126.2	70.7-197.7	
≥3 packs per week	21	25.1	14.7-38.3	0.26	19	35.2	21.3-52.7	0.98	19	100.8	63.2-147	0.28
Duration of use												
<14 years	13	24.3	12.1-40.8		11	37.5	20.1-60.3		11	121.7	71.0-186.1	
≥14 years	16	23.8	13.3-37.3	0.91	15	34.5	20.5-52.0	0.70	15	98.9	61.1-145.8	0.30
Alcohol use												
Never	5	16.8	7.6-29.6	ref	4	23.2	9.8-42.3	ref	4	102.6	59.0-158.3	ref
Former	8	16.9	8.1-28.9	0.99	8	22.9	11.0-39.1	0.98	8	108.5	68.6-157.5	0.83
Current user	46	14.1	9.4 19.8	0.58	43	20.4	13.7-28.4	0.70	43	128.7	103.1-157.1	0.30
Age at first use												
<18 years	20	12.1	6.9-18.8		17	17.7	10.2-27.2		17	138.9	106.0-176.2	
≥18 years	34	15.1	9.7-21.7	0.29	34	22.3	14.8-31.5	0.26	34	122.6	95.4-153.2	0.30
Drinks consumed												
<2 drinks per week	35	13.4	8.3-19.7		33	19.1	12.2-27.6		33	120.4	93.8-150.3	
≥2 drinks per week	19	14.9	8.8-22.5	0.62	18	23.4	14.6-34.3	0.32	18	143.5	110.2-181.2	0.14

^1^Adjusted least-square means, 95% confidence intervals, and *P*-values are from analyses using square root-transformed mammographic density measures. The adjusted least-square means and 95% confidence intervals were back-transformed

All models were adjusted for age (continuous) and BMI (continuous) at the time of mammogram, parity (continuous), and mammogram modality (digital image, film scanned by study team, film scanned by imaging centre). The height and weight models were mutually adjusted for each other (continuous) instead of BMI. The physical activity models were additionally adjusted for smoking status (never, former, current). The smoking models were additionally adjusted for the number of alcoholic drinks consumed per week (continuous). The alcohol models were additionally adjusted for smoking status (never/ever). TWA, total weekly activity. MVPA, moderate-to-vigorous physical activity. METs, Metabolic Equivalent of Task

### Lifestyle Risk Factors

Among premenopausal women who reported ever smoking (n = 33), increasing age of smoking initiation was associated with greater dense area (β = 0.30; 95% CI 0.08, 0.53; *P* = 0.01) (Additional file 3: Table S3). Increasing number of packs of cigarettes smoked per week was associated with significantly lower dense area (β = -0.41; 95% CI -0.77, -0.05; *P* = 0.03) (Additional file 3: Table S3). Further, those who reported smoking at least three cigarette packs per week had a lower adjusted mean dense area of 25.5 cm^2^ compared to 48.1 cm^2^ among women who smoked fewer packs per week (*P* = 0.01) (Table 4). Increasing duration of smoking was associated with significantly lower percent density (β = -0.07; 95% CI -0.13, -0.01; *P* = 0.02) and lower dense area (β = -0.12; 95% CI -0.18, -0.07; *P* = 0.0002) (Additional file 3: Table S3). Women who reported smoking for at least 14 years had a significantly lower adjusted mean percent density (15.8% vs. 32.3%; *P* = 0.01) and an adjusted mean dense area (25.3 cm^2^ vs. 53.1 cm^2^; *P* = 0.002) compared to women who smoked for a shorter duration (Table 4).

Among postmenopausal women who reported ever smoking (n = 29), former smokers had a significantly greater adjusted mean percent density (19.5% vs. 10.8%; *P* = 0.003), a greater adjusted mean dense area (26.9 cm^2^ vs. 16.4 cm^2^; *P* = 0.01) and a lower adjusted mean non-dense area (108.4 cm^2^ vs. 138.6 cm^2^; *P* = 0.04) compared to never smokers (Table 5). When this model was run continuously, former smokers were found to have significantly higher percent density (β = 1.13; 95% CI 0.41, 1.84; *P* = 0.003), higher dense area (β = 1.13; 95% CI 0.26, 2.00; *P* = 0.01), and lower non-dense area (β = -1.36; 95% CI -2.68, -0.04; *P* = 0.04) compared to never smokers (Additional file 4: Table S4).

Among premenopausal women, increasing age of first use of alcohol was associated with significantly higher percent density (β = 0.16; 95% CI 0.02, 0.31; *P* = 0.03) and dense area (β = 0.23; 95% CI 0.06, 0.40; *P* = 0.01) (Additional file 3: Table S3). Among postmenopausal women, increasing age of first use of alcohol was associated with higher percent density (β = 0.11; 95% CI 0.03, 0.19; *P* = 0.01) and dense area (β = 0.16; 95% CI 0.07, 0.25; *P* = 0.0006) (Additional file 4: Table S4).After applying the Bonferroni correction (new significance level: *P* ≤ 0.0002), the inverse associations between body weight and percent density and dense area remained significant among premenopausal women (*P* <0.0001 each). Among both premenopausal and postmenopausal women, the positive association between body weight and non-dense area remained significant (*P* <0.0001 each). In premenopausal women, the inverse association between duration of smoking and dense area remained significant (*P* = 0.0002). All other findings were non-significant based on the corrected *P-*value.

## Discussion

In this cross-sectional analysis of women with a strong family history of breast cancer and no *BRCA* mutation, various exposures were associated with mammographic density; although not all associations were in the directions that were previously reported among the general population. These findings suggest that mammographic density may act as an intermediate factor in the pathway between some of these risk factors and their influence on breast cancer risk in this cohort, particularly, parity, premenopausal body weight, and height. Although we examined a specific cohort of women, these results suggest similar mechanisms may mediate the relationships between some breast cancer risk factors and disease among this cohort and among the general population.

Parity was significantly associated with lower mammographic density among both premenopausal and postmenopausal women. These findings suggest that parity is associated with changes in both the dense and non-dense areas of the breast, and that mammographic density may mediate the influence of parity on breast cancer risk in this cohort. Parity is an established protective factor for breast cancer, and is thought to exert this effect on breast cancer risk by reducing the pool of mammary stem cells that are susceptible to genetic change [16, 17]. This biological hypothesis may explain why parous women typically have lower percent density compared to nulliparous women in the general population [17], as well as in our study population.

In the general population, a high body size is protective against breast cancer in premenopausal women, but is positively associated with breast cancer risk in postmenopausal women [18, 19]. Despite this paradoxical relationship, body size is inversely associated with percent mammographic density due to a positive association with non-dense area in both premenopausal and postmenopausal women [18]. Adiposity may result in an increased risk of breast cancer among postmenopausal women, where it is the primary source of endogenous estrogens, by stimulating the proliferation of dense epithelial cells in the breast [18, 20]. In the current study, body weight was inversely associated with percent mammographic density and positively associated with non-dense area among both premenopausal and postmenopausal women. These findings suggest that mammographic density may mediate the association between body weight and breast cancer in premenopausal women, but it likely does not mediate this association in postmenopausal women.

A greater adult attained height has previously been shown to be positively associated with percent mammographic density [3, 18, 21], and this is possibly due to the influence of endocrine hormones and circulating growth factors, such as IGF-1 and prolactin [21]. In our study, we did not observe an association between height and percent density and dense area. Interestingly, height was inversely associated with non-dense area in both premenopausal and postmenopausal women, suggesting that the positive relationship between height and percent density observed in other studies could also be a result of changes in adiposity, rather than changes in dense tissue.

Among premenopausal women, both an increasing duration of smoking and packs of cigarettes smoked per week were associated with lower percent density, while an increasing age of initiation of smoking was associated with greater dense area. Our results suggest an inverse association between smoking and mammographic density, which supports most [22–24], but not all [25, 26], of the existing literature. While this inverse relationship contradicts what is known about early exposure to smoking and breast cancer risk, many studies have reported positive associations between age of initiation of smoking and cigarettes smoked per day and mammographic density, in both premenopausal and postmenopausal cohorts [22–24]. Interestingly, we found that postmenopausal former smokers, but not current smokers, had higher adjusted mean percent density and dense area, and significantly lower non-dense area compared to never smokers. Similarly, two studies have observed higher percent density among both premenopausal and postmenopausal women who quit smoking compared to never smokers and current smokers [22, 23]. Based on these findings, the anti-estrogenic properties of tobacco may be associated with mammographic density, rather than its carcinogenic properties [22], and thus, mammographic density is unlikely to mediate the effect of smoking on breast cancer risk in this cohort.

Alcohol use is positively associated with mammographic density and with breast cancer risk in the general population [24, 25, 27–29], likely due to its stimulatory effect on circulating estrogens and IGF-1 levels, which may increase dense epithelial cell proliferation [28, 30]. In our study, an increasing age of initiation of alcohol was associated with significantly greater percent density and dense area among premenopausal and postmenopausal women. Premenopausal women who first consumed alcohol after age 18 had significantly lower adjusted mean non-dense area compared to women who consumed alcohol earlier. Overall, in contrast to others, our findings suggest an inverse association between alcohol consumption and mammographic density, suggesting that mammographic density likely does not mediate the association between alcohol and breast cancer risk in this cohort.

Strengths of the current study include the novelty of the study population, which was comprised of women with a strong family history of breast cancer but no identified *BRCA* mutation in their affected relatives. Our group previously estimated that this cohort faces a lifetime risk of breast cancer of 40% by age 70 [31]. Other prospective cohorts of women with a family history of breast cancer have been established [8, 32–34], but there is wide variation in their family history criteria, and *BRCA* mutation status is not consistently ascertained. While other studies have included non-carriers from *BRCA* mutation-positive families [35], these women may still represent a different risk profile than women from *BRCA* mutation-negative families. Therefore, our prospective cohort study based at an academic research hospital differs from other familial studies given our strict family history criteria, which considers both the relation of the affected relatives to the subject and their ages at diagnosis, and confirmation of negative *BRCA* mutation status [9]. Another strength to this study is the use of the Cumulus software to assess mammographic density, which performs well against qualitative and fully-automated methods, and is currently the gold standard for measuring mammographic density [14].

A primary limitation of our analysis was the small sample size, which may have limited the statistical power of our analyses, and therefore increased the likelihood of Type II error. As a result, we may have failed to observe a statistically significant association between some of the exposures of interest and the mammographic density measures. The sample size also limited our ability to test for effect modification by BMI and hormone replacement therapy use, which have been shown to be important when evaluating the associations between alcohol and mammographic density [25]. Due to the low number of smokers and nulliparous women in the sample, we could not evaluate the effects of smoking and alcohol before first full-term pregnancy on mammographic density, which may be a more important risk factor than age of initiation [22, 30]. Moreover, only six subjects reported ever using chemopreventive drugs, and only three have taken tamoxifen, therefore the relationship between chemoprevention and mammographic density could not be evaluated. A further limitation is that the mammograms and questionnaires were completed at different time points, with the premenopausal women having a significantly greater mean time difference compared to postmenopausal women (0.9 years vs. 0.6 years; *P* = 0.01), as well as a wide range of 0 to 5.6 years. However, the mean time differences in both groups were less than one year, which highlights the uptake of intensive breast screening among this high risk cohort. Since Cumulus is an area-based technique, it is unable to consider the 3-dimensional depth (i.e., volume) of the breast [36]. In addition, its reliance on a human reader is associated with the possibility of bias, although our reliability between readers was high (mean within-batch correlation coefficient: 0.91).

## Conclusions

In this preliminary cross-sectional analysis, various risk factors were associated with mammographic density among women with a strong family history of breast cancer. Future studies including a larger sample size and a longer follow-up are needed to determine the joint effects of these risk factors and mammographic density on breast cancer risk as the primary endpoint in this high-risk cohort. If replicated in larger studies, these findings may have important implications for counselling women from high-risk families about targeting modifiable risk factors to reduce mammographic density. Further evidence may also support the inclusion of mammographic density independently of other risk factors into breast cancer risk prediction models, which are used to identify women who will benefit from high-risk breast cancer screening and primary prevention strategies.

## Additional files

Additional file 1: **Table S1.** Difference in mammographic density measures according to reproductive and hormonal exposures among premenopausal women. (DOCX 19 kb)

Additional file 2: **Table S2.** Difference in mammographic density measures according to reproductive and hormonal exposures among postmenopausal women. (DOCX 20 kb)

Additional file 3: **Table S3.** Difference in mammographic density measures according to anthropometric and lifestyle factors among premenopausal women. (DOCX 20 kb)

Additional file 4: **Table S4.** Difference in mammographic density measures according to anthropometric and lifestyle factors among postmenopausal women. (DOCX 20 kb)

## Data Availability

The data analyzed during the current study are available upon reasonable request from the corresponding author.

## Abbreviations

BMI: body mass index
CI: confidence interval
DHQ: Diet History Questionnaire
IGF-1: insulin-like growth factor 1
MET: Metabolic Equivalent of Task
MVPA: moderate-to-vigorous physical activity

## References

[1] McCormack V. A. (2006). Breast Density and Parenchymal Patterns as Markers of Breast Cancer Risk: A Meta-analysis. Cancer Epidemiology Biomarkers & Prevention.

[2] Boyd NF, Martin LJ, Yaffe MJ, Minkin S (2011). Mammographic density and breast cancer risk: current understanding and future prospects. Breast Cancer Res.

[3] Martin LJ, Boyd NF (2008). Mammographic density. Potential mechanisms of breast cancer risk associated with mammographic density: hypotheses based on epidemiological evidence. Breast Cancer Res.

[4] Martin LJ, Melnichouk O, Guo H, Chiarelli AM, Hislop TG, Yaffe MJ, Minkin S, Hopper JL, Boyd NF (2010). Family history, mammographic density, and risk of breast cancer. Cancer Epidemiol Biomarkers Prev.

[5] Maskarinec G, Nakamura KL, Woolcott CG, Conroy SM, Byrne C, Nagata C, Ursin G, Vachon CM (2015). Mammographic density and breast cancer risk by family history in women of white and Asian ancestry. Cancer Causes Control.

[6] Crest AB, Aiello EJ, Anderson ML, Buist DS (2006). Varying levels of family history of breast cancer in relation to mammographic breast density (United States). Cancer Causes Control.

[7] Rice MS, Bertrand KA, VanderWeele TJ, Rosner BA, Liao X, Adami HO, Tamimi RM (2016). Mammographic density and breast cancer risk: a mediation analysis. Breast Cancer Res.

[8] Sung H, Ren J, Li J, Pfeiffer RM, Wang Y, Guida JL, Fang Y, Shi J, Zhang K, Li N (2018). Breast cancer risk factors and mammographic density among high-risk women in urban China. NPJ Breast Cancer.

[9] Kotsopoulos J, Metcalfe K, Alston J, Nikitina D, Ginsburg O, Eisen A, Demsky R, Akbari M, Zbuk K, Narod SA (2014). Prospective study of high-risk, BRCA1/2-mutation negative women: the 'negative study. BMC cancer.

[10] Csizmadi I, Kahle L, Ullman R, Dawe U, Zimmerman TP, Friedenreich CM, Bryant H, Subar AF (2007). Adaptation and evaluation of the National Cancer Institute's Diet History Questionnaire and nutrient database for Canadian populations. Public Health Nutr.

[11] Colditz GA, Hankinson SE (2005). The Nurses’ Health Study: lifestyle and health among women. Nat Rev Cancer.

[12] Wolf AM, Hunter DJ, Colditz GA, Manson JE, Stampfer MJ, Corsano KA, Rosner B, Kriska A, Willett WC (1994). Reproducibility and validity of a self-administered physical activity questionnaire. Int J Epidemiol.

[13] Ainsworth BE, Haskell WL, Leon AS, Jacobs DR, Montoye HJ, Sallis JF, Paffenbarger RS (1993). Compendium of physical activities: classification of energy costs of human physical activities. Med Sci Sports Exerc.

[14] Eng A, Gallant Z, Shepherd J, McCormack V, Li J, Dowsett M, Vinnicombe S, Allen S, dos-Santos-Silva I (2014). Digital mammographic density and breast cancer risk: a case-control study of six alternative density assessment methods. Breast Cancer Res.

[15] Byng JW, Boyd NF, Little L, Lockwood G, Fishell E, Jong RA, Yaffe MJ (1996). Symmetry of projection in the quantitative analysis of mammographic images. European journal of cancer prevention : the official journal of the European Cancer Prevention Organisation.

[16] Woolcott CG, Koga K, Conroy SM, Byrne C, Nagata C, Ursin G, Vachon CM, Yaffe MJ, Pagano I, Maskarinec G (2012). Mammographic density, parity and age at first birth, and risk of breast cancer: an analysis of four case-control studies. Breast Cancer Res Treat.

[17] Yaghjyan L, Colditz GA, Rosner B, Bertrand KA, Tamimi RM (2016). Reproductive factors related to childbearing and mammographic breast density. Breast Cancer Res Treat.

[18] Boyd NF, Martin LJ, Sun L, Guo H, Chiarelli A, Hislop G, Yaffe M, Minkin S (2006). Body size, mammographic density, and breast cancer risk. Cancer Epidemiol Biomarkers Prev.

[19] Harris HR, Tamimi RM, Willett WC, Hankinson SE, Michels KB (2011). Body size across the life course, mammographic density, and risk of breast cancer. American journal of epidemiology.

[20] Pettersson A, Hankinson SE, Willett WC, Lagiou P, Trichopoulos D, Tamimi RM (2011). Nondense mammographic area and risk of breast cancer. Breast Cancer Res.

[21] Lope V, Pérez-Gómez B, Moreno MP, Vidal C, Salas-Trejo D, Ascunce N, Román IG, Sánchez-Contador C, Santamariña MC, Carrete JA (2011). Childhood factors associated with mammographic density in adult women. Breast Cancer Res Treat.

[22] Butler LM, Gold EB, Conroy SM, Crandall CJ, Greendale GA, Oestreicher N, Quesenberry CP, Habel LA (2010). Active, but not passive cigarette smoking was inversely associated with mammographic density. Cancer Causes Control.

[23] Jacobsen KK, Lynge E, Vejborg I, Tjønneland A, von Euler-Chelpin M, Andersen ZJ (2016). Cigarette smoking and mammographic density in the Danish Diet, Cancer and Health cohort. Cancer Causes Control.

[24] Cabanes A, Pastor-Barriuso R, García-López M, Pedraz-Pingarrón C, Sánchez-Contador C, Vázquez Carrete JA, Moreno MP, Vidal C, Salas D, Miranda-García J (2011). Alcohol, tobacco, and mammographic density: a population-based study. Breast Cancer Res Treat.

[25] Brand JS, Czene K, Eriksson L, Trinh T, Bhoo-Pathy N, Hall P, Celebioglu F (2013). Influence of lifestyle factors on mammographic density in postmenopausal women. PLoS One.

[26] Gapstur SM, López P, Colangelo LA, Wolfman J, Van Horn L, Hendrick RE (2003). Associations of breast cancer risk factors with breast density in Hispanic women. Cancer Epidemiol Biomarkers Prev.

[27] Flom JD, Ferris JS, Tehranifar P, Terry MB (2009). Alcohol intake over the life course and mammographic density. Breast Cancer Res Treat.

[28] Ziembicki S, Zhu J, Tse E, Martin LJ, Minkin S, Boyd NF (2017). The Association between Alcohol Consumption and Breast Density: A Systematic Review and Meta-analysis. Cancer Epidemiol Biomarkers Prev.

[29] Liu Y, Nguyen N, Colditz GA (2015). Links between alcohol consumption and breast cancer: a look at the evidence. Womens Health (Lond).

[30] Liu Y, Colditz GA, Rosner B, Berkey CS, Collins LC, Schnitt SJ, Connolly JL, Chen WY, Willett WC, Tamimi RM (2013). Alcohol intake between menarche and first pregnancy: a prospective study of breast cancer risk. Journal of the National Cancer Institute.

[31] Metcalfe KA, Finch A, Poll A, Horsman D, Kim-Sing C, Scott J, Royer R, Sun P, Narod SA (2009). Breast cancer risks in women with a family history of breast or ovarian cancer who have tested negative for a BRCA1 or BRCA2 mutation. British journal of cancer.

[32] Terry MB, Phillips KA, Daly MB, John EM, Andrulis IL, Buys SS, Goldgar DE, Knight JA, Whittemore AS, Chung WK (2016). Cohort Profile: The Breast Cancer Prospective Family Study Cohort (ProF-SC). Int J Epidemiol.

[33] Sellers TA, Anderson VE, Potter JD, Bartow SA, Chen PL, Everson L, King RA, Kuni CC, Kushi LH, McGovern PG (1995). Epidemiologic and genetic follow-up study of 544 Minnesota breast cancer families: design and methods. Genet Epidemiol.

[34] Xu Z, Bolick SC, DeRoo LA, Weinberg CR, Sandler DP, Taylor JA (2013). Epigenome-wide association study of breast cancer using prospectively collected sister study samples. J Natl Cancer Inst.

[35] Ramón Y, Cajal T, Chirivella I, Miranda J, Teule A, Izquierdo Á, Balmaña J, Sánchez-Heras AB, Llort G, Fisas D, Lope V (2015). Mammographic density and breast cancer in women from high risk families. Breast Cancer Res.

[36] Destounis Stamatia, Arieno Andrea, Morgan Renee, Roberts Christina, Chan Ariane (2017). Qualitative Versus Quantitative Mammographic Breast Density Assessment: Applications for the US and Abroad. Diagnostics.

