# Supplementary material for: Predictors of mammographic density among women with a strong family history of breast cancer
Source: BMC Cancer. 2019 Jun 26;19:631. doi: 10.1186/s12885-019-5855-2 (PMC6595553; doi:10.1186/s12885-019-5855-2)
Supplement: Supplementary file 2 — Table S2. Difference in mammographic density measures according to reproductive and hormonal exposures among postmenopausal women. (DOCX 20 kb) [file 12885_2019_5855_MOESM2_ESM.docx]

Additional File 2: Table S2. Difference in mammographic density measures according to reproductive and hormonal exposures among postmenopausal women

|  |  | Postmenopausal women (*n* = 59) | | | | | | | | | |
| --- | --- | --- | --- | --- | --- | --- | --- | --- | --- | --- | --- |
|  |  | Percent Density (%) | |  |  | Dense Area (cm^2^) | |  |  | Non-Dense Area (cm^2^) | |
|  | *n* | β-Estimate (95% CI)^1^ | *P^1^* |  | *n* | β-Estimate (95% CI)^1^ | *P^1^* |  | *n* | β-Estimate (95% CI)^1^ | *P^1^* |
| Age at menarche, years | 59 | 0.05 (-0.22, 0.32) | 0.71 |  | 55 | 0.14 (-0.18, 0.46) | 0.37 |  | 55 | -0.06 (-0.53, 0.41) | 0.81 |
|  |  |  |  |  |  |  |  |  |  |  |  |
| Parity  Nulliparous  Parous | 13  46 | ref  -0.49 (-1.33, 0.34) | 0.24 |  | 11  44 | ref  -0.59 (-1.60, 0.42) | 0.25 |  | 11  44 | ref  0.36 (-1.14, 1.87) | 0.63 |
| Total live births^2^ | 46 | -0.27 (-0.79, 0.26) | 0.31 |  | 44 | -0.18 (-0.72, 0.36) | 0.51 |  | 44 | 0.53 (-0.41, 1.46) | 0.26 |
|  |  |  |  |  |  |  |  |  |  |  |  |
| Breastfeeding^2^  Never  Ever | 10  36 | ref  0.02 (-1.06, 1.10) | 0.97 |  | 10  34 | ref  -0.57 (-1.65, 0.5) | 0.29 |  | 10  34 | ref  -1.07 (-2.92, 0.79) | 0.25 |
| Breastfeeding duration, months^2^ | 46 | 0.02 (-0.03, 0.07) | 0.46 |  | 44 | 0.02 (-0.03, 0.07) | 0.45 |  | 44 | -0.04 (-0.13, 0.05) | 0.36 |
|  |  |  |  |  |  |  |  |  |  |  |  |
| OC use  Never  Ever | 5  53 | ref  0.73 (-0.51, 1.97) | 0.24 |  | 4  50 | ref  0.43 (-1.16, 2.02) | 0.59 |  | 4  50 | ref  -0.87 (-3.18, 1.44) | 0.45 |
| Duration of OC use, years | 53 | -0.15 (-0.91, 0.61) | 0.69 |  | 50 | 0.01 (-0.05, 0.08) | 0.73 |  | 50 | 0.04 (-0.06, 0.13) | 0.43 |
|  |  |  |  |  |  |  |  |  |  |  |  |
| HRT use  Never  Former  Current | 43  9  7 | ref  0.15 (-0.89, 1.19)  0.32 (-0.89, 1.52) | ref  0.77  0.60 |  | 39  9  7 | ref  -0.09 (-1.27, 1.09)  0.45 (-0.94, 1.83) | ref  0.88  0.52 |  | 39  9  7 | ref  -1.52 (-3.22, 0.19)  -0.41 (-2.40, 1.58) | ref  0.08  0.68 |

^1^β-Estimates, 95% confidence intervals, and *P*-values are from analyses using square root-transformed mammographic density measures.

^2^Among parous women only.

All models were adjusted for age (continuous) and BMI (continuous) at the time of mammogram, parity (continuous), and mammogram modality (digital image, film scanned by study team, film scanned by imaging centre). The parity (total live births) and breastfeeding models were additionally adjusted for age at first birth (continuous). OC, oral contraceptives. HRT, hormone replacement therapy.
