# Supplementary material for: Predictors of mammographic density among women with a strong family history of breast cancer
Source: BMC Cancer. 2019 Jun 26;19:631. doi: 10.1186/s12885-019-5855-2 (PMC6595553; doi:10.1186/s12885-019-5855-2)
Supplement: Supplementary file 3 — Table S3. Difference in mammographic density measures according to anthropometric and lifestyle factors among premenopausal women. (DOCX 20 kb) [file 12885_2019_5855_MOESM3_ESM.docx]

Additional File 3: Table S3. Difference in mammographic density measures according to anthropometric and lifestyle factors among premenopausal women

|  |  | Premenopausal women (*n* = 97) | | | | | | | | | |
| --- | --- | --- | --- | --- | --- | --- | --- | --- | --- | --- | --- |
|  |  | Percent Density (%) | |  |  | Dense Area (cm^2^) | |  |  | Non-Dense Area (cm^2^) | |
|  | *n* | β-Estimate (95% CI)^1^ | *P^1^* |  | *n* | β-Estimate (95% CI)^1^ | *P^1^* |  | *n* | β-Estimate (95% CI)^1^ | *P^1^* |
| Weight (kg) | 97 | -0.05 (-0.07, -0.03) | <0.0001 |  | 88 | -0.01 (-0.04, 0.02) | 0.38 |  | 88 | 0.13 (0.09, 0.17) | <0.0001 |
| Height (cm) | 97 | 0.04 (-0.01, 0.10) | 0.10 |  | 88 | 0.02 (-0.05, 0.08) | 0.66 |  | 88 | -0.12 (-0.21, -0.03) | 0.01 |
|  |  |  |  |  |  |  |  |  |  |  |  |
| TWA (MET-hrs/week) | 97 | 0.00 (-0.01, 0.01) | 0.95 |  | 88 | 0.00 (-0.01, 0.02) | 0.66 |  | 88 | -0.01 (-0.02, 0.01) | 0.51 |
| MVPA (MET-hrs/week) | 97 | 0.00 (-0.02, 0.01) | 0.70 |  | 88 | 0.00 (-0.02, 0.02) | 0.91 |  | 88 | -0.01 (-0.03, 0.02) | 0.54 |
|  |  |  |  |  |  |  |  |  |  |  |  |
| Smoking  Never  Former  Current | 64  26  7 | ref  0.10 (-0.71, 0.91)  -1.00 (-2.43, 0.42) | ref  0.81  0.17 |  | 58  23  7 | ref  0.23 (-0.85, 1.30)  -1.36 (-3.23, 0.52) | ref  0.68  0.15 |  | 58  23  7 | ref  0.66 (-0.76, 2.08)  -0.10 (-2.57, 2.38) | ref  0.36  0.94 |
| Age at first use | 33 | 0.18 (-0.03, 0.39) | 0.09 |  | 30 | 0.30 (0.08, 0.53) | 0.01 |  | 30 | -0.03 (-0.35, 0.28) | 0.84 |
| Packs smoked per week | 33 | -0.19 (-0.51, 0.13) | 0.23 |  | 30 | -0.41 (-0.77, -0.05) | 0.03 |  | 30 | 0.06 (-0.42, 0.54) | 0.79 |
| Duration of use (years) | 33 | -0.07 (-0.13, -0.01) | 0.02 |  | 30 | -0.12 (-0.18, -0.07) | 0.0002 |  | 30 | 0.01 (-0.09, 0.10) | 0.90 |
|  |  |  |  |  |  |  |  |  |  |  |  |
| Alcohol  Never  Former  Current | 10  15  72 | ref  0.10 (-1.36, 1.55)  -0.10 (-1.30, 1.09) | ref  0.90  0.87 |  | 10  15  72 | ref  -0.45 (-2.31, 1.42)  -0.28 (-1.95, 1.24) | ref  0.64  0.66 |  | 10  15  72 | ref  -1.23 (-3.64, 1.18)  -0.36 (-2.42, 1.69) | ref  0.31  0.73 |
| Age at first use | 87 | 0.16 (0.02, 0.31) | 0.03 |  | 79 | 0.23 (0.06, 0.40) | 0.01 |  | 79 | -0.19 (-0.44, 0.05) | 0.12 |
| Drinks per week | 87 | 0.02 (-0.10, 0.14) | 0.74 |  | 79 | 0.02 (-0.12, 0.16) | 0.80 |  | 79 | 0.04 (-0.16, 0.24) | 0.67 |

^1^β-Estimates, 95% confidence intervals, and *P*-values are from analyses using square root-transformed mammographic density measures.

All models were adjusted for age (continuous) and BMI (continuous) at the time of mammogram, parity (continuous), and mammogram modality (digital image, film scanned by study team, film scanned by imaging centre). The height and weight models were mutually adjusted for each other (continuous) instead of BMI. The physical activity models were additionally adjusted for smoking status (never, former, current). The smoking models were additionally adjusted for the number of alcoholic drinks consumed per week (continuous). The alcohol models were additionally adjusted for smoking status (never/ever). TWA, total weekly activity. METs, metabolic task equivalent. MVPA, moderate to vigorous physical activity.
